# *NOD1* and *NOD2* Genetic Variants in Association with Risk of Gastric Cancer and Its Precursors in a Chinese Population

**DOI:** 10.1371/journal.pone.0124949

**Published:** 2015-05-01

**Authors:** Zhe-Xuan Li, Yu-Mei Wang, Fu-Bing Tang, Lian Zhang, Yang Zhang, Jun-Ling Ma, Tong Zhou, Wei-Cheng You, Kai-Feng Pan

**Affiliations:** Key Laboratory of Carcinogenesis and Translational Research (Ministry of Education), Department of Cancer Epidemiology, Peking University Cancer Hospital & Institute, 52 Fu-cheng Road, Hai-dian District, Beijing 100142, China; Medical College of Soochow University, CHINA

## Abstract

**Background:**

Genetic variants of nucleotide-binding oligomerization domain-containing protein (NOD) may influence the outcome of *Helicobacter pylori* (*H*. *pylori*) infection and gastric carcinogenesis. To explore genetic variants of *NOD1* and *NOD2* in association with gastric cancer (GC) and its precursors, a population-based study was conducted in Linqu County, China.

**Methods:**

TagSNPs of *NOD1* and *NOD2* were genotyped by Sequenom MASS array in 132 GCs, and 1,198 subjects with precancerous gastric lesions, and were correlated with evolution of gastric lesions in 766 subjects with follow-up data.

**Results:**

Among seven tagSNPs, *NOD1* rs2709800 and *NOD2* rs718226 were associated with gastric lesions. *NOD1* rs2709800 TG genotype carriers had a decreased risk of intestinal metaplasia (IM, OR: 0.53; 95% CI: 0.31–0.92), while *NOD2* rs718226 G allele (AG/GG) showed increased risks of dysplasia (DYS, OR: 2.96; 95% CI: 1.86–4.71) and GC (OR: 2.35; 95% CI: 1.24–4.46). Moreover, an additive interaction between rs718226 and *H*. *pylori* was found in DYS or GC with synergy index of 3.08 (95% CI: 1.38–6.87) or 3.99 (95% CI: 1.55–10.22), respectively. The follow-up data indicated that *NOD2* rs2111235 C allele (OR: 0.52; 95% CI: 0.32–0.83) and rs7205423 G allele (OR: 0.56; 95% CI: 0.35–0.89) were associated with decreased risk of progression in *H*. *pylori*-infected subjects.

**Conclusions:**

*NOD1* rs2709800, *NOD2* rs718226, rs2111235, rs7205423 and interaction between rs718226 and *H*. *pylori* infection may be related to risk of gastric lesions.

## Introduction

Nearly one million new gastric cancers (GCs) occurred worldwide, 42% of them in China [1, 2]. Persistent *Helicobacter pylori* (*H*. *pylori*) infection induces gastric chronic inflammation, resulting in a transition from normal mucosa to chronic atrophic gastritis (CAG) followed by intestinal metaplasia (IM) and dysplasia (DYS), and subsequently increases the risk of GC [3–5]. Our previous intervention trials in Linqu County, a rural area with high mortality of GC in Shandong Province of China [6], indicated that *H*. *pylori* eradication could significantly reduce the risk of GC and its precursors [7–9]. Despite the high prevalence of *H*. *pylori* infection, only a small proportion of infected subjects eventually develop GC [10], suggesting that host genetic polymorphisms, especially in inflammation related genes may play pivotal roles in gastric carcinogenesis [11, 12].

The nucleotide-binding oligomerization domain (NOD)-like receptors (NLRs) belong to evolution-conserved pattern recognition receptors (PRRs) family locating in cytoplasm. Stimulated by exogenous pathogen-associated molecular patterns (PAMPs) or endogenous damage-associated molecular patterns (DAMPs) [13], NLRs may induce NF-κB and MAPK signaling pathways activation and expression of immune response cytokines and chemokines [14]. Activation of NLRs can drive innate immune response, regulate adaptive immune responses, and may also participate in carcinogenesis via regulating apoptosis [15, 16].

Two members of NLRs, namely NOD1 and NOD2, have been reported to be the key regulators of chronic infiammation induced by *H*. *pylori* infection [17, 18] and to be related to clinical outcome of *H*. *pylori* infection [18]. Although single nucleotide polymorphisms (SNPs) of *NOD1* and *NOD2* such as rs2066842 (P268S), rs2066844 (R702W), rs2066845 (G908R), and rs2066847 (L1007insC) have been found to be associated with risk of GC and precancerous lesions in Caucasian population [19–21], most of these SNPs were monomorphic in the Chinese population. Evidences on association between SNPs of *NOD1* and *NOD2* with risk of GC or precancerous lesions in Chinese population are limited. Although Wang et al. has correlated eight tagSNPs of *NOD1* and *NOD2* with risk of GC in a hospital-based case-control study [22], the relationship of genetic variants in *NOD1* and *NOD2*, precancerous gastric lesions and their evolution in the process of gastric carcinogenesis are yet unclear. Thus, the genome-wide association studies or tagSNP studies covering SNPs in the whole coding region of *NOD1* and *NOD2* in a Chinese population are required, especially in a high-risk population for GC.

In 1994 and 2002, we launched two randomized, intervention trials to inhibit the progression of gastric lesions and GC in Linqu County [7, 8]. Baseline data as well as follow-up information on population from placebo arms and untreated groups of these two cohorts have provided us a unique opportunity to explore the association between genetic polymorphisms *NOD1* and *NOD2* and evolution of gastric lesions, and possible interactions with *H*. *pylori* infection.

## Methods

### Study design and population

Two independent randomized intervention trials in Linqu County were launched in 1994 (Cohort I, registered in the U.S. National Cancer Institute PDQ database with trial number NCI-OH-95-C-N029; available at http://www.cancer.gov/clinicaltrials/) and 2002 (Cohort II, registered as HARECCTR0500053 in accordance with WHO ICTRP requirements) respectively [7, 8]. All participants underwent an endoscopic screening and provided blood samples at baseline, and repeated endoscopic examination with the same procedures at end of the trial. The detailed information of the study population, endoscopic procedures and criteria of gastric pathology had been described previously [7, 8]. Briefly, for each subject, biopsies were taken from the standard locations of stomach and histopathologic findings were evaluated by three senior pathologists from Peking University Cancer Hospital independently, according to the Updated Sydney System [23] and Padovo International Classification [24]. Each biopsy was classified according to the presence or absence of superficial gastritis (SG), CAG (mild or severe), IM, indefinite dysplasia (IndDYS), DYS or GC. Each subject was assigned a global diagnosis based on the most severe diagnosis among any of the biopsies. Baseline information on age, gender, cigarette smoking history, etc. for each participant was obtained by a standard structured questionnaire.

**Fig 1:**
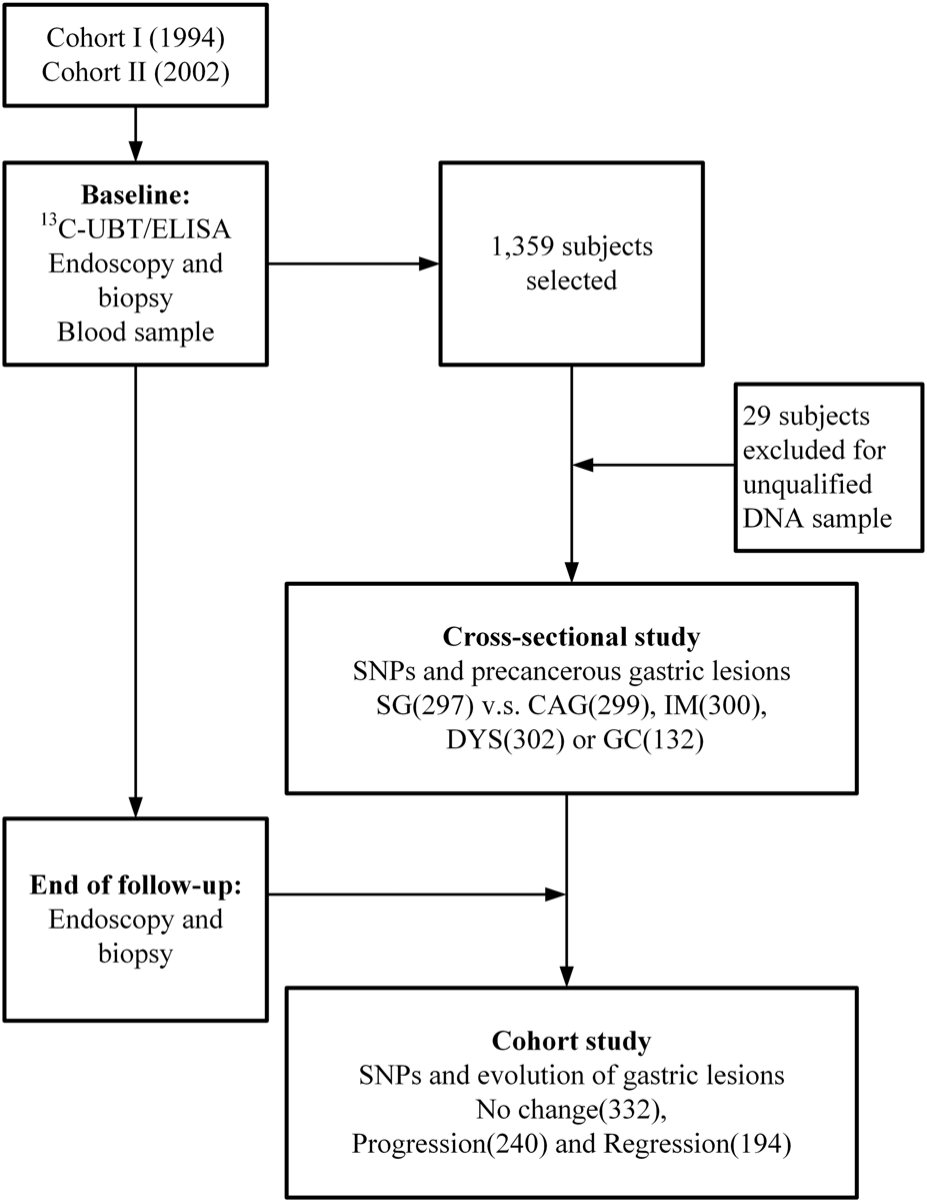
Diagram of study design.

For the current study, subjects with different precancerous gastric lesions at baseline including SG, CAG, IM, IndDYS, and DYS were selected at random from each pathology strata. To standardize both trials, subjects with precancerous lesions were further classified into SG (311), CAG (306), IM (300), and DYS (303). Additionally, a total of 139 GCs either identified from the baseline screening or during the follow-up period were enrolled. After genotyped in the selected 1,359 subjects, 29 of them were excluded for the low DNA concentration, subsequently, a total of 1,330 subjects were finally analyzed in this study. Among them, 766 subjects completing follow-up in the placebo arms of the two cohorts were selected to evaluate the association between genetic polymorphisms of *NOD1* or *NOD2* and evolution of *H*. *pylori*-associated gastric lesions (Fig 1). The study was approved by the Institutional Review Board of Peking University Cancer Hospital and all subjects gave written informed consent.

### Blood sample collection and DNA preparation

A 5-mL blood sample collected from each subject was allowed to clot for 30 to 40 minutes at room temperature and then centrifuged at 965 g for 15 minutes. The resulting serum was separated into vials. The clot and serum were frozen immediately at -20°C and stored in a -70°C freezer. High-molecular-weight genomic DNA was isolated from blood clot by standard proteinase-K digestion and phenol–chloroform extraction from the blood sample.

### *Helicobacter pylori* determination

*H*. *pylori* status was determined by enzyme-linked immunosorbent assay (ELISA) at baseline and by ^13^C-urea Breath Test (^13^C-UBT) after the anti-*H*. *pylori* treatment for cohort I and entire cohort II. Details of *H*. *pylori* antibody assay were described previously [25]. In brief, serum anti-*H*. *pylori* IgG and IgA levels were measured separately in duplicate with ELISA procedures. Quality control samples were assayed at Vanderbilt University, Nashville, TN. An individual was considered to be positive for *H*. *pylori* infection if the mean optical density for either the IgG or IgA >1.0, a cut-off value based on *H*. *pylori* negative persons and the reference sera.

For subjects from Cohort II, *H*. *pylori* status was determined by ^13^C-UBT at baseline in 2002. Details of the ^13^C-UBT were described in previous publications [26]. Briefly, after baseline samples of exhaled CO_2_ collection, participant was then requested to drink 20 ml of water with a pill of 80 mg ^13^C-urea (min. 99 atom % ^13^C). Exhaled CO_2_ was collected in sampling tubes 30 min later. ^13^CO_2_ values were determined by a gas isotopic ratio mass spectrometer, and any concentration of ^13^CO_2_ at 30 min that exceeded the baseline concentration by more than four parts per 1,000 (>0.4%) was regarded as a positive result.

### TagSNPs selection and genotyping

TagSNPs for the *NOD1* and *NOD2* genes were selected from the designable set of common SNPs with a minor allele frequency (MAF) over 0.05 genotyped in the Han Chinese (CHB) population samples of the HapMap Project (Hapmap Data Rel 27 PhaseII+III, Feb09, on NCBI B36 assembly, dbSNP b126). Applying Haploview software version 4.2 (available at: http://www.broadinstitute.org/scientific-community/science/programs/medical-and-population-genetics/haploview/) with pairwise linkage disequilibrium (LD) of r^2^ ≥ 0.80, 16 tagSNPs (7 tagSNPs for *NOD1* and 9 tagSNPs for *NOD2*) were initially enrolled in the pilot study, which covered the whole gene and 5k bp extension to 5’-flanking and 3’-flanking. For designing or Hardy-Weinberg equilibrium reasons, 2 tagSPNs (rs2709800 and rs2907749) capturing 28 out of 47 (59.5%) SNPs of *NOD1*, and 5 tagSNPs (rs718226, rs1077861, rs2111235, rs3135500, and rs7205423) capturing 18 out of 24 (75.0%) SNPs of *NOD2* were enrolled in the final analysis (S1 Table).

The genotypes were performed by KPS Biotechnology Co., Ltd (Beijing, China) by the Sequenom MassARRAY system (Sequenom, San Diego, CA, USA). A total of 10ng genomic DNA isolated from the peripheral blood lymphocytes of each study subjects was used for genotyping. Duplicated tests were performed in 5% of the samples gaining a concordance rate of 100%. Genotyping primers of all the seven SNPs were presented in S2 Table.

### Statistical methods

The Pearson’s *χ* ^2^ test was applied to compare the distribution of gender, smoking status, *H*. *pylori* infection among different gastric lesions or evolution groups, while Kruskal-Wallis or Wilcoxon test was used in testing age differences. The Hardy-Weinberg equilibrium of the genotype distribution was tested by the goodness-of-fit *χ* ^2^ test in reference group (SG subjects). The evolution status of gastric lesions for each subject was classified into progression group, no change group or regression group as described previously according to its global histopathologic diagnosis changes over baseline and end point [27].

Odds ratios (ORs) and 95% confidence intervals (CIs) were calculated by unconditional logistic regression to evaluate risks of gastric lesions or evolution associated with SNPs as well as gene-*H*. *pylori* joint effects adjusting for potential confounders. Multiplicative gene-*H*. *pylori* interactions were measured by including main effect variables and their product terms in the logistic regression model with likelihood ratio tests. And additive gene-*H*. *pylori* interactions were evaluated by the approximate variance estimation with synergy index and 95% CIs [28]. A value of *P* < 0.05 was considered statistically significant. All the statistical analyses were conducted by Statistical Analysis System software (version 9.2; SAS Institute, Cary, NC).

## Results

A total of 1,330 subjects (727 males and 603 females) with mean age of 48.54±8.69 years at baseline were enrolled in this study, including 297 subjects with SG, 299 with CAG, 300 with IM, 302 with DYS and 132 with GC, respectively. Of 766 subjects with follow-up data, 240 were progression, 194 were regression and 332 remained no change. The distributions of age, gender, smoking and *H*. *pylori* infection status were significantly different within gastric lesion groups (*P*<0.01). The mean age of GC cases was older than SG controls (53.83±9.89 vs. 47.56±8.32), and the proportions of male (76.52% vs. 58.25%), smoking (62.12% vs. 44.44%) and *H*. *pylori* infection (76.52% vs. 32.66%) were higher in GCs in contrast to SGs. For the cohort design, only *H*. *pylori* infection status showed statistical difference between evolution groups. Detailed information about the subjects was listed in Table 1.

**Table 1:** Selected characteristics and risk factors in the study subjects, (n, %).

Variables	Cross-sectional study, N = 1,330						Cohort study, N = 766					
	SG	CAG	IM	DYS	GC	*P* *	No progression	Progression	*P* *	No regression	Regression	*P* *
	N = 297	N = 299	N = 300	N = 302	N = 132		N = 526	N = 240		N = 572	N = 194	
Age (mean±SD)	47.56±8.32	45.56±7.23	48.56±7.88	50.11±9.23	53.83±9.89	<0.01**	47.85±8.11	47.38±8.74	0.25^†^	47.48±8.46	48.35±7.83	0.08^†^
Gender												
Female	124(41.75)	162(54.18)	162(54.00)	124(41.06)	31(23.48)	<0.01	259(49.24)	119(49.58)	0.93	282(49.30)	96(49.48)	0.96
Male	173(58.25)	137(45.82)	138(46.00)	178(58.94)	101(76.52)		267(50.76)	121(50.42)		290(50.70)	98(50.52)	
Smoking												
No	162(54.55)	187(62.54)	192(64.00)	137(45.36)	44(33.33)	<0.01	311(59.13)	134(55.83)	0.33	331(57.87)	114(58.76)	0.92
Yes	132(44.44)	111(37.12)	105(35.00)	159(52.65)	82(62.12)		211(40.11)	106(44.17)		237(41.43)	80(41.24)	
Missing	3(1.01)	1(0.33)	3(1.00)	6(1.99)	6(4.55)		4(0.76)	0(0)		4(0.70)	0(0)	
*H*. *pylori*												
Negative	200(67.34)	123(41.14)	69(23.00)	79(26.16)	27(20.45)	<0.01	215(40.87)	117(48.75)	0.03	270(47.20)	62(31.96)	<0.01
Positive	97(32.66)	175(58.53)	229(76.33)	223(73.84)	101(76.52)		311(59.13)	120(50.00)		299(52.27)	132(68.04)	
Unclear	0(0)	1(0.33)	2(0.67)	0(0)	4(3.03)		0(0)	3(1.25)		3(0.52)	0(0)	

*Pearson’s χ^2^ test

**Kruskal-Wallis test

^†^ Wilcoxon test

The associations between the seven tagSNPs of *NOD1* (rs2709800 and rs2907749) and *NOD2* (rs718226, rs1077861, rs2111235, rs3135500, and rs7205423) with risks of GC and its precursors were evaluated by unconditional logistic regression model adjusting for age, gender, smoking, and *H*. *pylori* infection status. Taking SG group as reference, risks for precancerous lesions and GC were negatively correlated with *NOD1* rs2709800 G allele, though only TG genotype in IM subjects reached statistical significance (OR: 0.53; 95% CI: 0.31–0.92). For *NOD2*, rs718226 AG genotype (OR: 2.50; 95% CI: 1.28–4.88) or combined AG+GG genotype was associated with risk of GC (OR: 2.35; 95% CI: 1.24–4.46). The G allele of rs718226 was also associated with risk of precancerous gastric lesions, however, statistical significance was only observed in DYS group. Subjects carrying AG genotype experienced a 2.79-fold higher risk of DYS (OR: 2.79; 95% CI: 1.71–4.55), and for subjects with GG genotype or G allele carriers, the risk of DYS was 3.33 (OR: 4.33; 95% CI: 1.38–13.60) or 1.96 (OR: 2.96; 95% CI: 1.86–4.17) times higher than controls respectively. (Table 2)

**Table 2:** Risk of precancerous gastric lesions and GC associated with *NOD1* and *NOD2* polymorphisms.

SNPs	Genotype	SG	CAG			IM			DYS			GC		
		N (%)	N (%)	OR (95%CI) *	*P* *	N (%)	OR (95%CI) *	*P* *	N (%)	OR (95%CI) *	*P* *	N (%)	OR (95%CI) *	*P* *
NOD1 rs2709800	TT	39(13.13)	41(13.71)	1.00		53(17.67)	1.00		50(16.56)	1.00		18(13.64)	1.00	
	TG	156(52.53)	153(51.17)	0.87(0.52–1.45)	0.59	127(42.33)	**0.53(0.31–0.92)**	**0.02**	139(46.03)	0.74(0.43–1.28)	0.28	69(52.27)	0.84(0.39–1.80)	0.65
	GG	102(34.34)	105(35.12)	0.94(0.55–1.62)	0.82	120(40)	0.78(0.44–1.37)	0.39	113(37.42)	0.89(0.50–1.57)	0.68	45(34.09)	0.70(0.31–1.58)	0.39
	TG+GG	258(86.87)	258(86.29)	0.90(0.55–1.47)	0.66	247(82.33)	0.63(0.38–1.06)	0.08	252(83.45)	0.80(0.47–1.34)	0.39	114(86.36)	0.79(0.38–1.64)	0.52
NOD1 rs2907749	AA	146(49.16)	157(52.51)	1.00		131(43.67)	1.00		154(50.99)	1.00		74(56.06)	1.00	
	AG	129(43.43)	115(38.46)	0.81(0.57–1.16)	0.25	143(47.67)	1.23(0.84–1.81)	0.29	122(40.4)	0.94(0.64–1.37)	0.11	44(33.33)	0.62(0.36–1.07)	0.08
	GG	22(7.41)	27(9.03)	1.16(0.62–2.20)	0.64	26(8.67)	1.15(0.58–2.30)	0.69	26(8.61)	1.27(0.64–2.51)	0.45	13(9.85)	1.05(0.42–2.62)	0.91
	AG+GG	151(50.84)	142(47.49)	0.86(0.62–1.21)	0.39	169(56.34)	1.22(0.84–1.76)	0.30	148(49.01)	0.98(0.69–1.41)	0.93	57(43.18)	0.68(0.41–1.14)	0.14
NOD2 rs718226	AA	246(82.83)	250(83.61)	1.00		277(92.33)	1.00		213(70.53)	1.00		98(74.24)	1.00	
	AG	42(14.14)	44(14.72)	1.36(0.84–2.23)	0.21	19(6.33)	1.05(0.55–2.00)	0.88	76(25.17)	**2.79(1.71–4.55)**	**<0.01**	31(23.48)	**2.50(1.28–4.88)**	**<0.01**
	GG	5(1.68)	5(1.67)	1.38(0.37–5.11)	0.63	3(1)	1.13(0.25–5.17)	0.87	12(3.97)	**4.33(1.38–13.60)**	**0.01**	3(2.27)	1.34(0.21–8.55)	0.75
	AG+GG	47(15.82)	49(16.39)	1.37(0.85–2.18)	0.19	22(7.33)	1.06(0.58–1.95)	0.85	88(29.14)	**2.96(1.86–4.71)**	**<0.01**	34(25.75)	**2.35(1.24–4.46)**	**<0.01**
NOD2 rs1077861	TT	196(65.99)	192(64.21)	1.00		188(62.67)	1.00		191(63.25)	1.00		83(62.88)	1.00	0.59
	AT	91(30.64)	89(29.77)	0.95(0.66–1.38)	0.21	100(33.33)	1.05(0.70–1.56)	0.59	97(32.12)	1.01(0.68–1.50)	0.11	45(34.09)	0.86(0.50–1.48)	0.97
	AA	8(2.69)	15(5.02)	1.74(0.69–4.37)	0.24	10(3.33)	1.41(0.59–4.04)	0.53	12(3.97)	2.36(0.87–6.41)	0.09	3(2.27)	0.90(0.16–5.20)	0.90
	AT+AA	99(33.33)	104(34.79)	1.02(0.71–1.46)	0.92	110(36.66)	1.08(0.73–1.58)	0.71	109(36.09)	1.11(0.76–1.62)	0.61	48(36.36)	0.87(0.51–1.47)	
NOD2 rs2111235	TT	163(54.88)	159(53.18)	1.00		148(49.33)	1.00		154(50.99)	1.00		67(50.76)	1.00	
	TC	106(35.69)	115(38.46)	1.05(0.73–1.51)	0.79	127(42.33)	1.22(0.82–1.80)	0.33	123(40.73)	1.15(0.78–1.69)	0.48	54(40.91)	0.96(0.56–1.63)	0.87
	CC	28(9.43)	25(8.36)	0.88(0.47–1.62)	0.67	25(8.33)	1.31(0.67–2.57)	0.43	25(8.28)	1.26(0.65–2.42)	0.49	11(8.33)	0.89(0.34–2.34)	0.82
	TC+CC	134(45.12)	140(46.82)	1.01(0.72–1.43)	0.94	152(50.66)	1.23(0.85–1.79)	0.27	148(49.01)	1.17(0.81–1.68)	0.40	65(49.24)	0.95(0.57–1.56)	0.83
NOD2 rs3135500	GG	188(63.30)	180(60.2)	1.00		176(58.67)	1.00		178(58.94)	1.00		74(56.06)	1.00	
	GA	91(30.64)	104(34.78)	1.17(0.81–1.681	0.41	105(35)	1.19(0.80–1.77)	0.40	106(35.1)	1.24(0.83–1.83)	0.29	48(36.36)	1.09(0.64–1.87)	0.75
	AA	18(6.06)	15(5.02)	0.92(0.43–1.97)	0.83	19(6.33)	1.33(0.62–2.87)	0.47	18(5.96)	1.38(0.64–2.96)	0.41	9(6.82)	1.30(0.46–3.67)	0.62
	GA+AA	109(36.70)	119(39.8)	1.13(0.80–1.60)	0.50	124(41.33)	1.21(0.83–1.76)	0.32	124(41.06)	1.26(0.87–1.82)	0.23	57(43.18)	1.12(0.68–1.87)	0.66
NOD2 rs7205423	CC	168(56.57)	161(53.85)	1.00		150(50)	1.00		155(51.32)	1.00		70(53.03)	1.00	
	CG	103(34.68)	121(40.47)	1.17(0.82–1.67)	0.40	122(40.67)	1.30(0.88–1.93)	0.19	122(40.4)	1.27(0.86–1.86)	0.23	50(37.88)	0.94(0.55–1.59)	0.81
	GG	26(8.75)	17(5.69)	0.66(0.33–1.31)	0.23	28(9.33)	1.44(0.74–2.77)	0.28	25(8.28)	1.17(0.61–2.25)	0.64	12(9.09)	0.96(0.38–2.45)	0.94
	CG+GG	129(43.43)	138(46.16)	1.07(0.76–1.50)	0.72	150(50)	1.33(0.92–1.92)	0.13	147(48.68)	1.25(0.87–1.79)	0.24	62(46.97)	0.94(0.57–1.55)	0.81

*Unconditional logistic regression adjusted for age, gender, smoking, and *H*. *pylori* infection status.

We further evaluated the potential joint effects or interactions between the *NOD1* rs2709800 or *NOD2* rs718226 and *H*. *pylori* infection on the risk of gastric lesions (Table 3). Since *NOD1* rs2709800 G allele was found associated with decreased risks of gastric lesions, we took *H*. *pylori* negative SG subjects carrying TG or GG genotype as reference. Risks of gastric lesions were significantly elevated in subjects with TT genotype and *H*. *pylori* infection, the ORs were 2.71 (95%CI: 1.27–5.78) for CAG, 11.06 (95%CI: 5.31–23.06) for IM, 8.16 (95%CI: 3.92–16.96) for DYS and reached 11.38 (95%CI: 4.04–32.06) for GC, respectively. Similarly, comparing with *NOD2* rs718226 AA genotype and *H*. *pylori* negative subjects, *H*. *pylori* infected G allele carriers experienced over 20 folds higher risks for DYS (OR: 25.23; 95% CI: 11.37–55.59) and GC (OR: 29.66; 95% CI: 11.18–78.69). An additive interaction between *NOD2* rs718226 G allele and *H*. *pylori* infection was found for DYS or GC, with synergy index of 3.08 (95% CI: 1.38–6.87) or 3.99 (95% CI: 1.55–10.22), respectively.

**Table 3:** Joint effect between polymorphisms and *H*. *pylori* infection on risk of precancerous gastric lesions and GC.

Polymorphisms	*H*. *pylori* infection	SG	CAG			IM			DYS			GC		
		N (%)	N (%)	OR (95%CI) *	*P* *	N (%)	OR (95%CI) *	*P* *	N (%)	OR (95%CI) *	*P* *	N (%)	OR (95%CI) *	*P* *
NOD1 rs2709800														
TG+GG	-	173(58.25)	103(34.56)	1.00		54(18.12)	1.00		65(21.52)	1.00		22(17.19)	1.00	
TG+GG	+	27(9.09)	20(6.71)	**2.80 (1.94–4.04)**	**<0.01**	15(5.03)	**8.31 (5.46–12.64)**	**<0.01**	14(4.64)	**7.00 (4.63–10.57)**	**<0.01**	5(3.91)	**10.25 (5.62–18.69)**	**<0.01**
TT	-	85(28.62)	154(51.68)	1.23 (0.65–2.34)	0.52	191(64.09)	1.90 (0.93–3.90)	0.08	187(61.92)	1.36 (0.65–2.85)	0.42	90(70.31)	1.55 (0.51–4.74)	0.44
TT	+	12(4.04)	21(7.05)	**2.71 (1.27–5.78)**	**0.01**	38(12.75)	**11.06 (5.31–23.06)**	**<0.01**	36(11.92)	**8.16 (3.92–16.96)**	**<0.01**	11(8.59)	**11.38 (4.04–32.06)**	**<0.01**
NOD2 rs718226														
AA	-	159(54.27)	87(29.19)	1.00		47(15.82)	1.00		50(16.61)	1.00		22(17.19)	1.00	
AA	+	38(12.97)	36(12.08)	**3.18 (2.18–4.62)**	**<0.01**	22(7.41)	**10.19 (6.63–15.67)**	**<0.01**	29(9.63)	**7.22 (4.64–11.22)**	**<0.01**	5(3.91)	**7.97 (4.31–14.74)**	**<0.01**
AG+GG	-	87(29.69)	162(54.36)	**1.72 (1.01–2.94)**	**0.04**	228(76.77)	**2.11 (1.12–3.98)**	**0.02**	163(54.15)	**2.66 (1.46–4.84)**	**<0.01**	74(57.81)	1.223 (0.42–3.60)	0.72
AG+GG	+	9(3.07)	13(4.36)	2.11 (0.85–5.22)	0.11	0(0)	NA	0.98	59(19.6)	**25.23 (11.37–55.99)**	**<0.01**	27(21.09)	**29.66 (11.18–78.69)**	**<0.01**

*Unconditional logistic regression adjusted for age, gender, and smoking.

Relative risk of progression or regression with the seven tagSNPs independently compared with no progression (no change and regression) or no regression (no change and progression) subjects were detected. As shown in Table 4, adjusting for age, gender, smoking, *H*. *pylori* status, and baseline pathology, *NOD2* rs2111235 and rs7205423 were associated with the risk of progression. Subjects carrying rs2111235 TC genotype (OR: 0.64; 95% CI: 0.44–0.92) or C allele (OR: 0.71; 95% CI: 0.50–0.99), and subjects with rs7205423 CG genotype (OR: 0.69; 95% CI: 0.48–0.98) had decreased risks of progression. However, no association between these tagSNPs with regression was found.

**Table 4:** Association between polymorphisms and risk of evolution of gastric lesions.

Polymorphisms	Progression				Regression			
	No, n (%)	Yes, n (%)	OR (95%CI) *	*P* *	No, n (%)	Yes, n (%)	OR (95%CI) *	*P* *
NOD1 rs2709800								
TT	95(18.06)	37(15.42)	1.00		101(17.66)	31(15.98)	1.00	
TG	246(46.77)	110(45.83)	1.04(0.64–1.70)	0.88	260(45.45)	96(49.48)	1.46(0.87–2.46)	0.15
GG	185(35.17)	93(38.75)	1.41(0.85–2.33)	0.18	211(36.89)	67(34.54)	1.08(0.63–1.85)	0.79
TG+GG	431(81.94)	203(84.58)	1.19(0.75–1.88)	0.46	471(82.34)	163(84.02)	1.27(0.78–2.08)	0.34
NOD1 rs2907749								
AA	254(48.29)	117(48.75)	1.00		281(49.13)	90(46.39)	1.00	
AG	228(43.35)	101(42.08)	1.07(0.75–1.53)	0.70	243(42.48)	86(44.33)	1.10(0.75–1.61)	0.63
GG	44(8.37)	21(8.75)	1.38(0.75–2.54)	0.31	47(8.22)	18(9.28)	1.07(0.56–2.04)	0.85
AG+GG	272(51.72)	122(50.83)	1.12(0.80–1.57)	0.51	290(50.7)	104(53.61)	1.09(0.76–1.57)	0.67
NOD2 rs718226								
AA	455(86.5)	213(88.75)	1.00		496(86.71)	172(88.66)	1.00	
AG	59(11.22)	24(10)	0.81(0.44–1.48)	0.49	63(11.01)	20(10.31)	1.12(0.57–2.22)	0.74
GG	10(1.9)	2(0.83)	0.55(0.11–2.88)	0.48	11(1.92)	1(0.52)	0.18(0.02–1.57)	0.12
AG+GG	69(13.12)	26(10.83)	0.78(0.44–1.39)	0.40	74(12.93)	21(10.83)	0.92(0.48–1.79)	0.81
NOD2 rs1077861								
TT	329(62.55)	160(66.67)	1.00		360(62.94)	129(66.49)	1.00	
AT	172(32.7)	70(29.17)	0.79(0.54–1.14)	0.21	184(32.17)	58(29.9)	0.84(0.57–1.25)	0.39
AA	19(3.61)	8(3.33)	0.91(0.37–2.27)	0.84	23(4.02)	4(2.06)	0.45(0.15–1.42)	0.17
AT+AA	191(36.31)	78(32.5)	0.80(0.56–1.14)	0.22	207(36.19)	62(31.96)	0.80(0.54–1.17)	0.24
NOD2 rs2111235								
TT	255(48.48)	137(57.08)	1.00		298(52.1)	94(48.45)	1.00	
TC	231(43.92)	79(32.92)	**0.64(0.44–0.92)**	**0.01**	224(39.16)	86(44.33)	1.18(0.81–1.72)	0.39
CC	40(7.6)	24(10)	1.11(0.60–2.04)	0.74	50(8.74)	14(7.22)	0.88(0.43–1.77)	0.71
TC+CC	271(51.52)	103(42.92)	**0.71(0.50–0.99)**	**0.04**	274(47.9)	100(51.55)	1.13(0.78–1.62)	0.52
NOD2 rs3135500								
GG	306(58.17)	151(62.92)	1.00		345(60.31)	112(57.73)	1.00	
GA	190(36.12)	75(31.25)	0.80(0.56–1.15)	0.23	193(33.74)	72(37.11)	1.11(0.76–1.63)	0.59
AA	30(5.7)	14(5.83)	0.97(0.46–2.02)	0.93	34(5.94)	10(5.15)	0.85(0.38–1.90)	0.69
GA+AA	220(41.82)	89(37.08)	0.82(0.58–1.16)	0.27	227(39.68)	82(42.26)	1.07(0.74–1.55)	0.72
NOD2 rs7205423								
CC	260(49.43)	139(57.92)	1.00		307(53.67)	92(47.42)	1.00	
CG	228(43.35)	82(34.17)	**0.69(0.48–0.98)**	**0.04**	223(38.99)	87(44.85)	1.25(0.86–1.83)	0.25
GG	38(7.22)	19(7.92)	1.18(0.61–2.28)	0.61	42(7.34)	15(7.73)	0.93(0.46–1.88)	0.84
CG+GG	266(50.57)	101(42.09)	0.75(0.53–1.05)	0.09	265(46.33)	102(52.58)	1.19(0.83–1.72)	0.34

*Unconditional logistic regression adjusted for age, gender, smoking, *H*. *pylori* infection status, and baseline pathology.

The risks of progression related to the two SNPs were further examined with stratification by baseline pathology or *H*. *pylori* infection status. Since a few subjects were SG (N = 55) in this cohort study, and only 6 out of 144 DYS subjects eventually progressed, we investigated the correlations between tagSNPs and risks of progression in CAG and IM strata. In CAG group, comparing with *NOD2* rs2111235 TT genotype, the risk of progression was about half decreased in subjects with TC genotype (OR: 0.51; 95% CI: 0.30–0.89) or C allele (OR: 0.58; 95% CI: 0.35–0.90). Risk of progression was only addressed in *H*. *pylori* positive group. In *H*. *pylori* positive subjects, *NOD2* rs2111235 C allele (TC+CC) and *NOD2* rs7205423 G allele (CG+GG) were associated with a decreased risk of progression, the ORs were 0.52 (95%CI: 0.32–0.83) for rs2111235 and 0.56 (95%CI: 0.35–0.89) for rs7205423, respectively (Table 5).

**Table 5:** Risk of progression of gastric lesions associated with polymorphisms stratified by baseline pathology or *H*. *pylori* infection status.

Polymorphisms	Progression vs. No progression in different baseline pathology strata						Progression vs. No progression in different *H*. *pylori* infection strata					
	CAG, N = 261			IM, N = 306			*H*. *pylori* positive, N = 431			*H*. *pylori* negative, N = 332		
	n/n	OR (95%CI) *	*P* *	n/n	OR (95%CI) *	*P* *	n/n	OR (95%CI) **	*P* **	n/n	OR (95%CI) **	*P* **
NOD2 rs2111235												
TT	64/72	1.00		48/104	1.00		70/142	1.00		65/113	1.00	
TC	33/70	**0.51(0.30–0.89)**	**0.02**	29/100	0.60(0.34–1.06)	0.08	37/142	**0.46(0.28–0.76)**	**<0.01**	41/89	0.88(0.52–1.50)	0.65
CC	11/11	1.00(0.40–2.53)	0.99	7/18	1.04(0.39–2.77)	0.93	13/27	0.82(0.37–1.82)	0.63	11/13	1.57(0.59–4.16)	0.37
TC+CC	44/81	**0.58(0.35–0.90)**	**0.04**	36/118	0.66(0.39–1.13)	0.13	50/169	**0.52(0.32–0.83)**	**0.01**	52/102	0.97(0.59–1.61)	0.91
NOD2 rs7205423												
CC	63/77	1.00		48/106	1.00		69/143	1.00		68/117	1.00	
CG	37/69	0.60(0.35–1.03)	0.06	29/95	0.65(0.37–1.15)	0.14	42/138	**0.53(0.32–0.87)**	0.01	39/90	0.85(0.50–1.45)	0.56
GG	8/7	1.33(0.44–3.98)	0.61	7/21	0.97(0.37–2.53)	0.94	9/30	0.68(0.29–1.64)	0.39	10/8	2.78(0.91–8.46)	0.07
CG+GG	45/76	0.67(0.40–1.12)	0.12	36/116	0.70(0.41–1.19)	0.19	51/168	**0.56(0.35–0.89)**	**<0.01**	49/98	0.99(0.60–1.65)	0.98

*Unconditional logistic regression adjusted for age, gender, smoking, and *H*. *pylori* infection status.

**Unconditional logistic regression adjusted for age, gender, smoking, and baseline pathology.

## Discussion

In this population-based study, we investigated the association between a panel of seven tagSNPs in *NOD1* and *NOD2* genes with the risks of GC and precancerous gastric lesions as well as their evolution. To our best knowledge, this is the first study exploring the impacts of genetic polymorphisms of NLR genes on the risk of gastric lesions and evolution of gastric lesions in a Chinese population at high risk of GC.

NOD1 and NOD2 expressed in epithelial and antigen-presenting cell recognizing peptidoglycan-derived peptides specifically [29, 30], may be crucial in *H*. *pylori* related processes of inflammation and carcinogenesis [15, 18, 31–33]. Over expression of NOD1 and NOD2 were found in human cell lines challenged with *H*. *pylori* cag PAI-positive strains [34, 35]. NOD1 activation might be essential for induction of both NF-κB and AP-1 activation during *H*. *pylori* infection by involving in translocation NF-κB and AP-1 complexes to the nucleus [18], whereas NOD2 was required for induction of inflammation cytokine pro-IL-1β in *H*. *pylori-*infected cells [36]. Hence, polymorphisms in these two genes might influence consequences of *H*. *pylori* induced chronic inflammation and further relate to risks of gastric lesions.

In this study, an intron variant rs2709800 of *NOD1* was found to be moderately associated with risk of gastric lesions. Subjects with TG genotype were at a low risk of IM comparing to TT genotype. Although no functional studies on this polymorphism were reported before, it was predicted that the G allele variant might be related to nonsense-mediated mRNA decay (NMD) which may cause low expression of NOD1. Therefore, the G allele carriers may be more likely at a low NOD1 expression status after infected by *H*. *pylori*, which may result in low activation of NF-κB mediated inflammation in gastric epithelia and consequently may relate to lower risk of gastric lesions than T allele carriers. However, the associations demands to be validated in functional studies. A nonsynonymous variant named rs2075820 (E266K) in *NOD1* gene was initially detected in our pilot study. However, as no statistical significance was observed, it was excluded for further investigation in this study.

Though several functional variants in *NOD2* such as rs2066842 (P268S), rs2066844 (R702W), rs2066845 (G908R), and rs2066847 (L1007insC) were studied in Caucasian population [19–21], these SNPs were reported monomorphic in the Chinese population in HapMap database. In our study, we applied a tagSNP strategy covering the whole sequence of *NOD2* and found rs718226 AG or GG genotype associated with increased risks of GC or DYS. The rs718226 located in 3’ near region of *NOD2*, probably participated in post transcription regulation. Further study should be conducted to reveal the functional relevance.

Considering the potentially functional relevance between *NOD1* or *NOD2* and *H*. *pylori* infection [29], we were also interested in possible interaction and joint effect between *NOD1* or *NOD2* polymorphism and *H*. *pylori* infection. We observed significant joint effects of *H*. *pylori* infection and *NOD1* rs2709800 or *NOD2* rs718226 on the risk of gastric lesions and an additive interaction between *NOD2* rs718226 and *H*. *pylori* infection. Although the precise mechanism on the *H*. *pylori*-associated carcinogenesis is unclear, it may be reasonable that these polymorphisms could affect post transcriptional regulation of NOD2, ligand recognition, or activation of innate immune response [29, 37]. Consequently increased secretion of pro-inflammatory cytokines due to persistent *H*. *pylori* infection, amplified the chronic inflammation and ultimately contributed to the development of GC.

Intestinal type GC is believed as an end result of multistage transition of gastric mucosa over years under chronic inflammation stress and CAG is a relatively common *H*. *pylori*-associated inflammatory condition and may be important in gastric carcinogenesis [38]. In this study, *NOD2* rs2111235 TC genotype was found decreasing the risk of progression in CAG subjects and in *H*. *pylori* infected subjects. The rs2111235 is an intron variant and C allele is predicted associated with NMD, which might decrease the expression of *NOD2* and secretion of pro-inflammatory cytokines via NF-κB pathway after *H*. *pylori* infection. Therefore, CAG subjects with rs2111235 C allele might have lower inflammation intensity after *H*. *pylori* infection and lower risk of progression.

The SNP of *NOD2* rs7205423 is located in the intergenic region between the *NOD2* and the *CYLD* gene, a de-ubiquitinating enzyme inhibiting the activation of NF-κB [39, 40]. Our study observed that *NOD2* rs7205423 G allele was negatively correlated with risk of progression in *H*. *pylori* infected subjects, which was in line with another study comparing GC cases with controls in a Chinese population [22]. The G allele of rs7205423 may be at a splice site [22], however further functional studies are needed. Two SNPs named rs2907749 and rs3135500 were also found to be associated with the risk of GC previously [22], but these two SNPs did not gain a statistical difference between GCs and controls in the present study. The study population selected from cohort study in an area at high risk of GC might contribute to the difference. In addition, differences in pathogenesis of diffuse and intestinal types of GC may be also a possible explanation to the inconsistency. The association between rs2907749 and risk of GC was only addressed in diffuse type cases in Wang’s study [22] whereas the majority of GC cases were found intestinal type in our previous studies in Linqu County [41]. Consequently, another validating study covering more GC cases is required.

Taking advantage of long-term follow-up cohort, we systematically analyzed SNPs in *NOD1* and *NOD2* associated with the risk of GC. This population-based study design allowed us to investigate the SNPs in a spectrum of precancerous gastric lesions and to explore the relationship between the SNPs and evolution of gastric lesions in the process of GC development. However, our study also has some limitations. Because there were very few subjects with normal gastric mucosa in the population, we only selected subjects with SG as controls which may cause an underestimated association between SNPs and risk of gastric lesions. Besides, restricting to 132 GC cases detected from our previous cohorts in an area at high risk of GC increased the homogeneity of study population. However, the relative small sample size of GC in this study calls for an independent case-control study including a more appropriate control group as well as more GC cases. Likewise, subjects with follow-up data in this study were only selected from placebo arm without intervention, which made it unavailable to analyze potential interactions between treatments, such as garlic supplementation, and SNPs on the progression of gastric lesions. Moreover, as we took the tagging SNPs strategy, the functional relevance of the newly identified polymorphisms including *NOD2* rs718226, rs2111235 as well as rs7205423 on the *H*. *pylori*-associated gastric carcinogenesis is limited and needs to be further clarified.

In summary, in this population-based study we found *NOD1* rs2709800 and *NOD2* rs718226 were associated with risk of precancerous gastric lesion or GC. In addition, significant additive interaction between *NOD2* rs718226 and *H*. *pylori* infection was observed, suggesting that genetic and environmental factors contribute to the etiology of GC together. Our study also found two SNPs in *NOD2* (rs2111235 and rs7205423) were correlated with progression risk of gastric lesions, particularly in *H*. *pylori* infected population, indicating these SNPs might serve as potential risk markers in GC prevention practices. Further studies in a larger sample size together with functional studies are warranted to confirm these findings.

## Supporting Information

S1 Table Tag and captured SNPs in NOD1 and NOD2 genes. (DOCX)

S2 Table Genotyping primers for SNPs in NOD1 and NOD2 genes. (DOCX)
