# Supplementary material for: NOD1 and NOD2 Genetic Variants in Association with Risk of Gastric Cancer and Its Precursors in a Chinese Population
Source: PLoS One. 2015 May 1;10(5):e0124949. doi: 10.1371/journal.pone.0124949 (PMC4416772; doi:10.1371/journal.pone.0124949)
Supplement: S1 Table — (DOCX) [file pone.0124949.s001.docx]

S1 Table. Tag and captured SNPs in NOD1 and NOD2 genes

| NOD1 | | |  | NOD2 | | |
| --- | --- | --- | --- | --- | --- | --- |
| Tag SNPs | Captured SNPs | R-square |  | Tag SNPs | Captured SNPs | R-square |
| rs10277025 | rs10277025 | 1 |  | rs1861757 | rs1861759 | 1 |
| rs17159048 | rs17159048 | 1 |  |  | rs1861757 | 1 |
| rs2284357 | rs2284357 | 1 |  | rs2111235 | rs2111235 | 1 |
| rs2907749 | rs2907749 | 1 |  |  | rs2111234 | 0.896 |
|  | rs2391869 | 0.934 |  | rs4785224 | rs4785224 | 1 |
|  | rs2284358 | 0.87329 |  |  | rs2067085 | 1 |
|  | rs1558066 | 0.934 |  | rs5743270 | rs5743270 | 1 |
|  | rs1558069 | 0.856478 |  | rs718226 | rs718226 | 1 |
|  | rs1558070 | 0.934 |  | rs7205423 | rs7205423 | 1 |
|  | rs4272257 | 0.934 |  | rs1077861 | rs6500328 | 0.839 |
|  | rs4363092 | 0.934 |  |  | rs8057341 | 0.839 |
|  | rs4722986 | 0.934 |  |  | rs13339578 | 0.839 |
|  | rs4722987 | 0.872356 |  |  | rs11642646 | 1 |
|  | rs6948524 | 0.934 |  |  | rs17312836 | 1 |
|  | rs732038 | 0.934 |  |  | rs11647841 | 0.839 |
|  | rs17159124 | 0.934 |  |  | rs4785225 | 0.821 |
|  | rs4720003 | 0.86395 |  |  | rs751271 | 0.839 |
|  | rs730361 | 0.86862 |  |  | rs748855 | 1 |
|  | rs730360 | 0.934 |  |  | rs1861758 | 1 |
|  | rs2893375 | 0.934 |  |  | rs10521209 | 1 |
|  | rs41524946 | 0.934 |  |  | rs1077861 | 1 |
|  | rs4722988 | 0.934 |  |  | rs3135499 | 0.888 |
|  | rs4720004 | 0.934 |  | rs3135500 | rs3135500 | 1 |
| rs2709803 | rs2075821 | 0.885 |  | rs8056611 | rs8056611 | 1 |
|  | rs2075820 | 0.945 |  |  |  |  |
|  | rs2235099 | 0.893 |  |  |  |  |
|  | rs2075819 | 1 |  |  |  |  |
|  | rs2075818 | 0.945 |  |  |  |  |
|  | rs2906766 | 0.846 |  |  |  |  |
|  | rs736781 | 0.937 |  |  |  |  |
|  | rs2709803 | 1 |  |  |  |  |
|  | rs2037955 | 0.944 |  |  |  |  |
|  | rs2709799 | 0.802 |  |  |  |  |
|  | rs2736726 | 0.846 |  |  |  |  |
| rs2970498 | rs10267377 | 0.939 |  |  |  |  |
|  | rs2907748 | 1 |  |  |  |  |
|  | rs2970498 | 1 |  |  |  |  |
|  | rs2970499 | 1 |  |  |  |  |
|  | rs2970500 | 1 |  |  |  |  |
| rs2709800 | rs7789045 | 0.909 |  |  |  |  |
|  | rs1558068 | 0.909 |  |  |  |  |
|  | rs2906773 | 0.909 |  |  |  |  |
|  | rs2529440 | 1 |  |  |  |  |
|  | rs2709801 | 0.909 |  |  |  |  |
|  | rs2709800 | 1 |  |  |  |  |
|  | rs932272 | 1 |  |  |  |  |
|  | rs2256023 | 0.953 |  |  |  |  |
