# Supplementary material for: NOD1 and NOD2 Genetic Variants in Association with Risk of Gastric Cancer and Its Precursors in a Chinese Population
Source: PLoS One. 2015 May 1;10(5):e0124949. doi: 10.1371/journal.pone.0124949 (PMC4416772; doi:10.1371/journal.pone.0124949)
Supplement: S2 Table — (DOCX) [file pone.0124949.s002.docx]

S2 Table. Genotyping primers for SNPs in NOD1 and NOD2 genes

| Gene | rs number | PCR 1st primer | PCR 2nd primer | Amplification length(bp) | Extension sequence primer |
| --- | --- | --- | --- | --- | --- |
| NOD1 | rs2709800 | ACGTTGGATGTCACACACACAGAAAGCCAG | ACGTTGGATGGCCCAGAAAGGCAAATTCTC | 117 | ATGAGTGAGGTGTGGCT |
| NOD1 | rs2907749 | ACGTTGGATGTGAAAAGAACAGCAAGGCCC | ACGTTGGATGAATCTCTGAGGTTGGGTGAG | 109 | TGGGTGAGTAGAAGGGGA |
| NOD2 | rs718226 | ACGTTGGATGTTGCTTCTGGGTGGAGATAG | ACGTTGGATGTCTCACCTGCAATAGCTCTG | 114 | GGCAGCCTCCCCACT |
| NOD2 | rs1077861 | ACGTTGGATGTCACATGAGTTAGCCCAGCG | ACGTTGGATGCCATAAGCAGGAAACAGGAC | 114 | GAAGGGGCATTTCTGA |
| NOD2 | rs2111235 | ACGTTGGATGTTGTCCTCATGAAGTCAGCC | ACGTTGGATGATTTTACCTGCCTGGCTACC | 97 | TTTTTAGGGGAAATCCCA |
| NOD2 | rs3135500 | ACGTTGGATGAAGTTCACGGCCATGTTGTC | ACGTTGGATGTATGATGTGTGAAAACTGG | 110 | TGTGAAAACTGGTTAATATTTATAG |
| NOD2 | rs7205423 | ACGTTGGATGTCCAGCCCATTTTGGACTTC | ACGTTGGATGTATGGCTGCTGCAGGAAATG | 93 | CACAAATTATCCCCTTATAGTC |
